# Comparison of Use and Appreciation of a Print-Delivered Versus CD-ROM-Delivered, Computer-Tailored Intervention Targeting Saturated Fat Intake: Randomized Controlled Trial

**DOI:** 10.2196/jmir.940

**Published:** 2008-04-29

**Authors:** Willemieke Kroeze, Anke Oenema, Marci Campbell, Johannes Brug

**Affiliations:** ^3^EMGO InstituteVU University Medical CenterAmsterdamThe Netherlands; ^2^Department of NutritionSchool of Public HealthUniversity of North CarolinaChapel HillNCUSA; ^1^Department of Public HealthErasmus MCUniversity Medical CenterRotterdamThe Netherlands

**Keywords:** Computer, Internet, tailoring, process evaluation, demographic differences, diet, nutrition education

## Abstract

**Background:**

Computer-tailored health education, a promising health education technique, is increasingly being delivered interactively, for example, over the Internet. It has been suggested that there may be differences in use and appreciation between print and interactive delivery of computer-tailored interventions, which may influence information processing. This may especially be the case for women, older people, and people of lower socioeconomic status. Knowledge about differences in use and appreciation could help in choosing the appropriate delivery mode for a particular target audience.

**Objective:**

The study investigates a content-identical, computer-tailored intervention addressing saturated fat intake delivered via print or CD-ROM. We analyzed consumer use and appreciation of the feedback information and explored whether possible differences exist among gender, age, and education subgroups.

**Methods:**

Healthy Dutch adults (18-65 years), none of whom were under treatment for hypercholesterolemia, were randomly allocated to receive a computer-tailored program on CD-ROM (n = 151) or in print (n = 141). At baseline, data were collected on gender, age, and education level. One month post-intervention, data were collected on the use (feedback information read, saved, discussed) and appreciation (trustworthiness, perceived individualization, perceived personal relevance, and user-friendliness) of the feedback. Statistical analyses on the use and appreciation items were performed using chi-square tests and independent-samples *t* tests.

**Results:**

After exclusion of individuals with missing values, a total of 257 and 240 respondents were included in the analyses of the use outcomes of feedback read and saved, respectively. The results indicate that among the total population, the print feedback was read more often than the CD-ROM feedback (95% vs 81%; *P* = .001) and saved more often than the CD-ROM feedback (97% vs 77%; *P* < .001). Similar results were found among the gender, age, and education subgroups. After exclusion of individuals who did not read the information and those with missing values, a total of 208-223 respondents were included in the analyses of the use outcome of feedback discussed and the appreciation items. The personal relevance of the print feedback was rated higher than for the CD-ROM-delivered feedback (0.97 vs 0.68; *P* = .04), but the effect size was small (0.28). These differences in personal relevance were also seen among women (1.06 vs 0.67; *P* = .04) and respondents aged 35-49 years (1.00 vs 0.58; *P* = .03), with moderate effect sizes (0.38 and 0.44, respectively).

**Conclusions:**

Despite the possible advantages of interactive feedback, the present study indicates that interactive-delivered feedback was used less and perceived as less personally relevant compared to the print-delivered feedback. These differences in use and appreciation of delivery modes should be taken into consideration when selecting a delivery mode for a specific subgroup in order to optimize exposure.

**Trial Registration:**

ISRCTN 01557410; http://www.webcitation.org/5XMylWleH

## Introduction

### Computer-Tailored Health Education

Computer-tailored health education delivers individualized information matched to an individual’s characteristics [1,2] and is a promising health education technique, particularly for (print-delivered) nutrition education [3]. The Internet is increasingly being used for the delivery of computer-tailored interventions. There are many features that make the Internet an attractive medium of delivery, such as the instant and continuous availability, the possibilities for interactivity, and the possibility to provide immediate feedback [4,5]. Another potential advantage is that larger numbers of people can be reached for lower cost, as compared with print-delivered interventions [4,6,7].

There also may be disadvantages of providing computer-tailored interventions over the Internet: it may be more difficult to read or process information from a computer screen [8,9], it may require more effort to receive the computer-tailored feedback (ie, starting the computer and the program), and people may be less likely to save and re-read interactive-delivered feedback [8]. Furthermore, it has been suggested that specific groups, such as people of lower socioeconomic status, women, and older people, will not be reached with interventions over the Internet because they may have more difficulty with and less interest in using interactive media [10-14].

On the other hand, some previous studies have shown that persons from lower socioeconomic groups have more interest in computer-tailored feedback compared to generic information [15-17]. In addition, the possibility of incorporating multiple mediums on the Internet to convey the information could reinforce comprehension for less-educated individuals [18]. Even though it has been suggested that there may be differences in use between print- and interactive-delivered computer-tailored interventions, the evidence to demonstrate this is still limited. The aim of the present study is to examine differences in use and appreciation of an identical-content, print-delivered versus interactive-delivered, computer-tailored intervention.

Knowledge about differences in use and appreciation could help in choosing the appropriate delivery mode for a particular target audience.

### Information Processing and Delivery Mode

Use and appreciation of an intervention are important factors to study since they are prerequisites for active information processing [19,20]. Active information processing is necessary for finally achieving changes in determinants and behavior [20]. Information processing starts with attention to the message [19], which can be operationalized as reading the information. The channel through which the information is provided is one of the factors that may determine attention to the message [19]. Attention to the message may be more easily achieved when the information is provided in a readily readable format or when it is provided via a medium that the receiver likes or knows how to use [13,21].

Active information processing not only involves attention to the message, but also thoughtful consideration of the information content. Reading, saving, and discussing the information with others may be indicators of active information processing. Furthermore, information is more likely to be attended to and actively processed when it is perceived as interesting, personally relevant, and individualized [22-26]. In a study by Oenema and colleagues, perceived personal relevance and individualization were identified as mediators of the effect of a computer-tailored intervention [26].

Even though, based on theory, indicators of use and perception of personal relevance are important for achieving intervention effects, these factors may be different for print and interactive deliveries: the medium may determine attention and access to the message [19], as well as the ability and willingness to actively process the information [13,27].

Only two previous studies have compared use of print- and Internet-delivered interventions with identical content [28,29]. Both studies reported higher recall and use of the print materials compared to the materials delivered through the Internet. In the current study we will evaluate a broader set of indicators for use and appreciation and perceptions of personal relevance between a print-delivered and an interactive-delivered, computer-tailored, nutrition education intervention with identical content aimed at reducing saturated fat intake. These interventions were found to be equally effective in reducing saturated fat intake in the short term, but only the effects of the print-delivered tailored feedback were maintained in the longer term [30].

The current study specifically examines whether there are differences in use (information read, saved, discussed with others) and appreciation (perceived personal relevance, perceived individualization, trustworthiness, user-friendliness) between print computer-tailored advice and interactive computer-tailored advice. These differences were examined for a mixed population and for gender, age, and education subgroups. A CD-ROM was used to deliver the interactive, Web-based intervention, enabling people who did not have Internet access to use the program.

**Figure 1:**
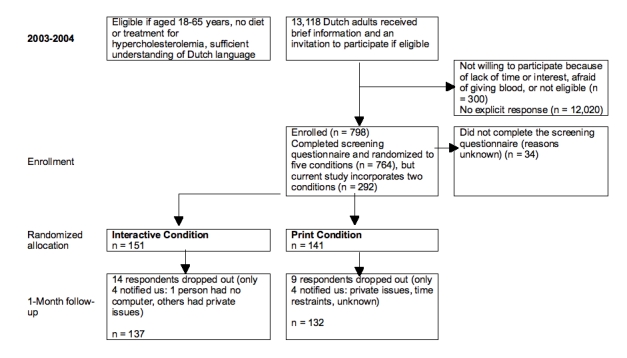
Subject recruitment and retention flowchart

## Methods

### Design and Recruitment

This study is part of a larger randomized controlled trial with five study arms. The current study uses data from two of the study arms for secondary data analysis: computer-tailored dietary saturated fat reduction feedback delivered on CD-ROM (n = 151) or delivered in print (n = 141). Approval for the research project was obtained from the Medical Ethics Committee of Erasmus University Medical Center, Rotterdam, The Netherlands. All participants gave written informed consent after receiving written information about the study. Volunteers for the larger intervention trial were recruited from among employees of nine large companies and inhabitants of two neighborhoods in the Rotterdam area (2003-2004). A total of 798 adults volunteered to participate, none of whom were on a prescribed diet or under treatment for hypercholesterolemia. Participants completed a baseline paper-and-pencil screening questionnaire and were subsequently randomized by computer to one of the two intervention conditions (Figure 1).

### Computer-Tailored Interventions

The tailored feedback in the current study incorporated feedback on personal saturated fat intake, social-comparison information, motivational feedback, practical product feedback addressing the most important sources of fat in the person’s diet, information on low-fat alternatives, and self-efficacy-enhancing feedback for difficult situations as derived from an individual assessment. The content of the computer-tailored program (screening questionnaire and feedback) was identical for the two intervention conditions, only the delivery mode was different, as described below. Details of the computer-tailored feedback are described elsewhere [30].

### CD-ROM Condition

**Figure 2:**
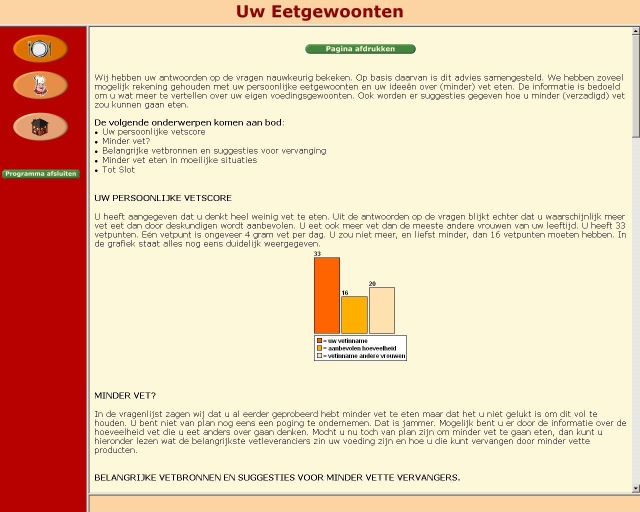
Example of part of the feedback delivered on CD-ROM

In the CD-ROM condition, the computer-tailored feedback was programmed as a series of Web pages (questionnaire, feedback messages), then stored on a CD-ROM. The program started with a home page explaining the nature and goal of the program and how it should be used. Immediately after completion of the screening questionnaire, the individualized computer-tailored information appeared on screen (Figure 2). Low-fat recipes for appetizers, main courses, and desserts could be searched from a recipe page. It was possible to print and save the feedback, but the program did not automatically do this. Respondents were asked to use the program on a computer with Internet Explorer 5.0 or higher and to use it in the same week they received the CD-ROM.

### Print Condition

**Figure 3:**
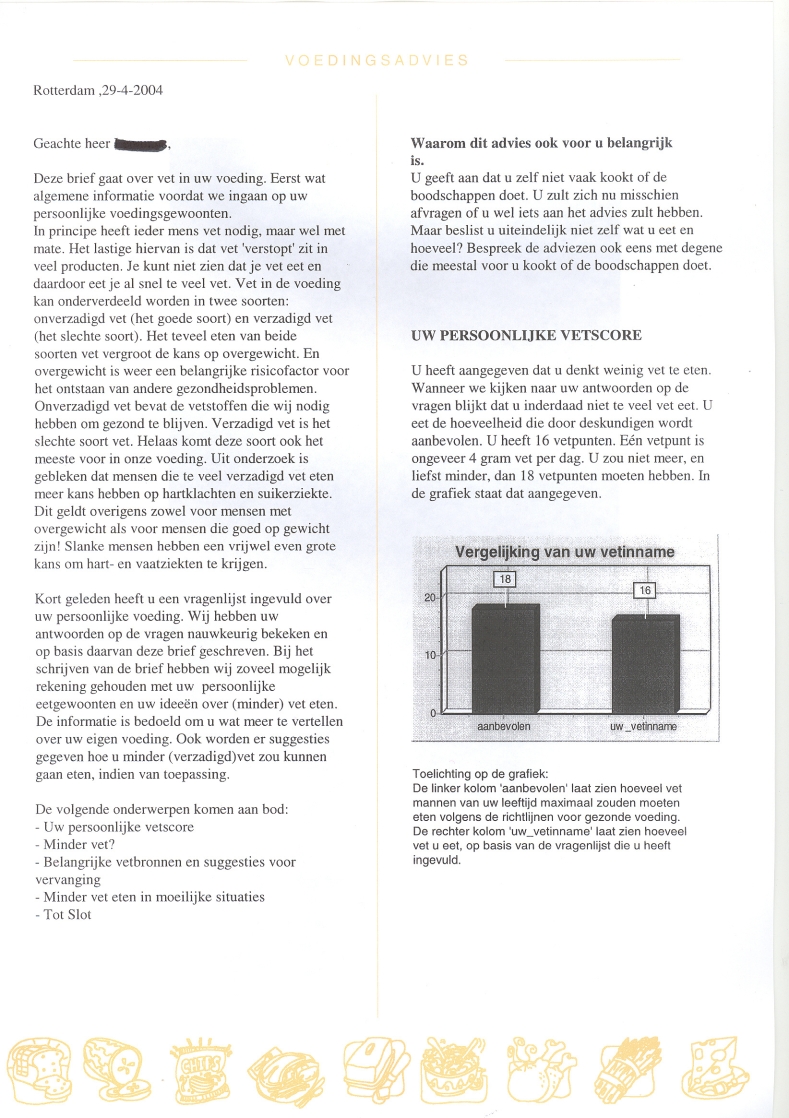
Example of part of the feedback delivered in print

The tailored information in the print condition was generated from the results of a baseline paper-and-pencil questionnaire. The questionnaires were scanned and imported into a computer-tailoring program that generated individualized computer-tailored printed feedback letters of 1.5-4 pages (Figure 3). Depending on their preferences, respondents received recipe suggestions for low-fat appetizers, main courses, or desserts. The feedback letters were sent to the home address of the respondent within 2 weeks of the time the study team received the completed questionnaire.

### Measurements

Gender, age, and education level were assessed in the baseline questionnaire. A categorical variable was created from age (≤ 34 years, 35-49 years, 50-65 years) [13]. Highest level of completed formal education was measured using one question in which seven education categories were distinguished (from elementary school to university degree) [31]. The categories were then collapsed into a three-level education variable (lower = lower secondary education or less; medium = upper secondary or post-secondary non-tertiary education; higher = college or university training).

At 1 month post-intervention, the outcome measures (use and appreciation items) were assessed. The questions were introduced by explaining that nutrition advice referred to either the advice delivered by a printed letter or by CD-ROM. Use was assessed with the following yes/no items: “I have read the complete nutrition advice”; “I saved the nutrition advice”; and “I discussed the nutrition advice with others.” Appreciation was assessed using a 5-point scale (from −2 = strongly disagree to +2 = strongly agree): “I perceived the nutrition advice as trustworthy”; “The nutrition advice addressed my personal dietary habits”; “The nutrition advice was of personal relevance for me”; and “The nutrition advice was user-friendly.” Appreciation questions were adapted from the process questionnaire as proposed by Brug and colleagues [23] and have been successfully used in previous studies [25,26].

### Statistical Analysis

Equality of the study groups at baseline was examined with chi-square tests (gender, education) and an independent-samples *t* test (age). Differences in use and appreciation outcomes between the intervention conditions were analyzed with chi-square tests (feedback read, saved, and discussed) and independent-samples *t* tests (trustworthy, perceived individualization, personal relevance, and user-friendliness). Respondents with missing values were excluded from the analyses. The “discussed” variable and the appreciation items were analyzed only for those respondents who confirmed they had read all the information. Finally, in order to compare the size of the difference in appreciation items between the print and the CD-ROM group, we calculated the effect sizes as the standardized differences in group means by dividing the difference between the conditions by the pooled standard deviation. Effect sizes were categorized as small (0-0.32), moderate (0.33-0.55), or large (> 0.55) as defined by Lipsey [32]. The analyses were performed for the total group, and, based on the literature [13], we decided a priori to conduct stratified analyses in specific subgroups based on gender, age, and education category. All analyses were conducted in SPSS version 11 (SPSS Inc, Chicago, IL, USA).

## Results

### Population Characteristics

Among the respondents (n = 292), 46% were male, the mean age was 43.9 years (SD 10.3), 22% fell in the lower education level, 34.4% in the medium education level, and 43.6% in the higher education level. There were no significant differences in gender, age, or education level between the two conditions.

### Use of the Computer-Tailored Information

As shown in Table 1, the print-delivered feedback was read more often than the CD-ROM-delivered feedback according to self-reports among the total population (*P* = .001) and among women (*P* = .003). This was also the case for participants in the 50-65 year age group (*P* = .01; Table 2) and for participants in lower and higher education levels (*P* = .04 for both groups; Table 3).

The print-delivered feedback was reported to be saved more often than the CD-ROM-delivered feedback among the total population (*P* < .001), men (*P* = .02), women (*P* < .001), the ≤ 34 year and 35-49 year age groups (*P* = .001 for both groups), and medium- and higher-educated respondents (*P* = .001 for both groups).

Less than 50% of those who reported to have read the tailored information discussed it with others.

**Table 1:** Use and appreciation of the print- and CD-ROM-delivered, computer-tailored intervention, by gender

	Total Study Group				Men				Women			
**Use (yes/no)**	**Print,No. (%)**	**CD-ROM,No. (%)**	P^*^	***χ*^2^_1_**	**Print,No. (%)**	**CD-ROM,No. (%)**	P^*^	***χ*^2^_1_**	**Print,No. (%)**	**CD-ROM,No. (%)**	P^*^	***χ*^2^_1_**
Read^†^	122/129 (94.6)	103/128 (80.5)	.001	11.73	53/58 (91)	45/57 (79)	.06	3.53	69/71 (97)	58/71 (82)	.003	9.02
Saved^†^	120/124 (96.8)	89/116 (76.7)	< .001	21.42	51/55 (93)	40/52 (77)	.02	5.25	69/69 (100)	49/64 (77)	< .001	18.23
Discussed^‡^	50/114 (43.9)	38/94 (40)	.62	0.25	25/48 (52)	16/40 (40)	.26	1.28	25/66 (38)	22/54 (41)	.75	0.10
												
**Appreciation (−2 to +2)**	**Mean ± SD**	**Mean ± SD**	***P***^§^	**ES^||^**	**Mean ± SD**	**Mean ± SD**	***P***^§^	**ES^||^**	**Mean ± SD**	**Mean ± SD**	***P***^§^	**ES^||^**
Trustworthy^‡^	1.28 ± 0.96 (n=120)	1.26 ± 1.02 (n=102)	.89	0.02	1.34 ± 0.88 (n=53)	1.33 ± 0.90 (n=45)	.97	0.01	1.24 ± 1.03 (n=67)	1.21 ± 1.11 (n=57)	.88	0.03
Perceived individualization^‡^	1.12 ± 1.01 (n=121)	1.01 ± 1.08 (n=102)	.45	0.11	1.04 ± 0.98 (n=53)	1.13 ± 0.97 (n=45)	.63	−0.09	1.18 ± 1.04 (n=68)	0.91 ± 1.15 (n=57)	.18	0.25
Personal relevance^‡^	0.97 ± 0.98 (n=119)	0.68 ± 1.11 (n=102)	.04	0.28	0.85 ± 1.02 (n=52)	0.69 ± 1.10 (n=45)	.47	0.15	1.06 ± 0.95 (n=67)	0.67 ± 1.12 (n=57)	.04	0.38
User-friendly^‡^	0.99 ± 1.07 (n=121)	1.09 ± 1.04 (n=102)	.50	−0.09	1.00 ± 1.06 (n=53)	1.33 ± 0.88 (n=45)	.10	−0.34	0.99 ± 1.09 (n=68)	0.89 ± 1.11 (n=57)	.65	0.09

^*^ *P* value derived from Pearson chi-square test.

^†^Only cases without missing values are included in analyses; therefore, numbers in denominators differ from numbers in Figure 1.

^‡^For the analysis of the variables discussed, trustworthy, perceived individualization, personal relevance, and user-friendly, only respondents who indicated they had read the information and without missing values were included in the analysis.

^§^ *P* value derived from independent-samples *t* test.

^||^Positive effect size (ES) in favor of print; negative ES in favor of CD-ROM; ES can be categorized as small (0-0.32), moderate (0.33-0.55), or large (> 0.55).

**Table 2:** Use and appreciation of the print- and CD-ROM-delivered, computer-tailored intervention, by age group

	≤ 34 Years				35-49 Years				50-65 Years			
**Use (yes/no)**	**Print,No. (%)**	**CD-ROM,No. (%)**	P^*^	***χ*^2^_1_**	**Print,No. (%)**	**CD-ROM,No. (%)**	P^*^	***χ*^2^_1_**	**Print,No. (%)**	**CD-ROM,No. (%)**	P^*^	***χ*^2^_1_**
Read^†^	22/23 (96)	26/30 (87)	.27	1.23	64/69 (93)	44/54 (82)	.06	3.60	36/37 (97)	33/44 (75)	.01	7.92
Saved^†^	22/22 (100)	17/30 (57)	.001	12.71	65/66 (99)	40/51 (78)	.001	12.57	33/36 (92)	32/35 (91)	.97	0.00
Discussed^‡^	8/20 (40)	10/25 (40)	1.00	0.00	30/61 (49)	17/40 (43)	.51	0.43	12/33 (36)	11/29 (38)	.90	0.02
												
**Appreciation (−2 to +2)**	**Mean ± SD**	**Mean ± SD**	***P***^§^	**ES^||^**	**Mean ± SD**	**Mean ± SD**	***P***^§^	**ES^||^**	Mean ± SD	**Mean ± SD**	***P***^§^	**ES^||^**
Trustworthy^‡^	1.18 ± 1.01 (n=22)	1.31 ± 0.97 (n=26)	.66	−0.13	1.35 ± 0.90 (n=63)	1.23 ± 1.02 (n=43)	.54	0.13	1.23 ± 1.06 (n=35)	1.27 ± 1.10 (n=33)	.87	−0.04
Perceived individualization^‡^	1.05 ± 1.13 (n=22)	0.88 ± 1.24 (n=26)	.64	0.14	1.22 ± 0.91 (n=63)	1.05 ± 1.00 (n=43)	.35	0.18	0.97 ± 1.11 (n=36)	1.06 ± 1.06 (n=33)	.74	−0.08
Personal relevance^‡^	0.81 ± 1.03 (n=21)	0.35 ± 1.23 (n=26)	.18	0.40	1.00 ± 0.94 (n=62)	0.58 ± 0.96 (n=43)	.03	0.44	1.00 ± 1.04 (n=36)	1.06 ± 1.12 (n=33)	.82	−0.06
User-friendly^‡^	0.68 ± 1.21 (n=22)	1.23 ± 1.03 (n=26)	.10	−0.49	1.00 ± 1.03 (n=63)	0.98 ± 0.99 (n=43)	.91	0.02	1.17 ± 1.03 (n=36)	1.12 ± 1.11 (n=33)	.86	0.05

^*^ *P* value derived from Pearson chi-square test.

^†^Only cases without missing values are included in analyses; therefore, numbers in denominators differ from numbers in Figure 1. ^‡^For the analysis of the variables discussed, trustworthy, perceived individualization, personal relevance, and user-friendly, only respondents who indicated they had read the information and without missing values were included in the analysis.

^§^ *P* value derived from independent-samples *t* test.

^||^Positive effect size (ES) in favor of print; negative ES in favor of CD-ROM; ES can be categorized as small (0-0.32), moderate (0.33-0.55), or large (> 0.55).

**Table 3:** Use and appreciation of the print- and CD-ROM-delivered, computer-tailored intervention, by education level

	Lower Education				Medium Education				Higher Education			
**Use (yes/no)**	**Print,No. (%)**	**CD-ROM,No. (%)**	P^*^	***χ*^2^_1_**	**Print,No. (%)**	**CD-ROM,No. (%)**	P^*^	***χ*^2^_1_**	**Print,No. (%)**	**CD-ROM,No. (%)**	P^*^	***χ*^2^_1_**
Read**^†^**	29/30 (967)	22/28 (79)	.04	4.47	43/46 (94)	35/43 (81)	.08	3.00	49/42 (94)	46/57 (81)	.04	4.45
Saved**^†^**	27/28 (96)	23/25 (92)	.49	0.49	46/46 (100)	31/39 (80)	.001	10.42	47/50 (94)	35/52 (67)	.001	11.52
Discussed^‡^	10/25 (40)	8/18 (44)	.77	0.09	20/43 (47)	13/32 (41)	.61	0.26	20/46 (44)	17/44 (39)	.64	0.22
												
**Appreciation (−2 to +2)**	**Mean ± SD**	**Mean ± SD**	***P***^§^	**ES^||^**	**Mean ± SD**	**Mean ± SD**	***P***^§^	**ES^||^**	**Mean ± SD**	**Mean ± SD**	***P***^§^	**ES^||^**
Trustworthy^‡^	1.29 ± 1.01 (n=28)	0.95 ± 1.33 (n=22)	.32	0.29	1.37 ± 0.82 (n=43)	1.34 ± 0.68 (n=35)	.87	0.04	1.20 ± 1.06 (n=49)	1.36 ± 1.07 (n=45)	.49	−0.15
Perceived individualization^‡^	1.14 ± 1.06 (n=29)	0.82 ± 1.14 (n=22)	.31	0.29	1.23 ± 0.84 (n=43)	1.11 ± 0.90 (n=35)	.55	0.14	1.00 ± 1.12 (n=49)	1.02 ± 1.18 (n=45)	.93	−0.02
Personal relevance^‡^	1.18 ± 1.02 (n=28)	0.77 ± 1.15 (n=22)	.19	0.39	1.07 ± 0.70 (n=42)	0.94 ± 0.91 (n=35)	.49	0.16	0.76 ± 1.13 (n=49)	0.42 ± 1.20 (n=45)	.17	0.29
User-friendly^‡^	1.14 ± 1.03 (n=29)	1.00 ± 1.19 (n=22)	.66	0.13	1.07 ± 0.99 (n=43)	1.17 ± 1.01 (n=35)	.66	−0.10	0.84 ± 1.16 (n=49)	1.07 ± 0.99 (n=45)	.31	−0.21

^*^ *P* value derived from Pearson chi-square test.

^†^Only cases without missing values are included in analyses; therefore, numbers in denominators differ from numbers in Figure 1. ^‡^For the analysis of the variables discussed, trustworthy, perceived individualization, personal relevance, and user-friendly, only respondents who indicated they had read the information and without missing values were included in the analysis.

^§^ *P* value derived from independent-samples *t* test.

^||^Positive effect size (ES) in favor of print; negative ES in favor of CD-ROM; ES can be categorized as small (0-0.32), moderate (0.33-0.55), or large (> 0.55).

### Appreciation of the Computer-Tailored Information

Trustworthiness, perceived individualization, and user-friendliness were not significantly different between the print condition and the CD-ROM condition. However, the CD-ROM condition was rated as more user-friendly by men (*P* = .10) and respondents ≤ 34 years (*P* = .10), with a moderate, though not statistically significant, effect size.

Results showed a statistically significant higher perceived personal relevance for the print condition compared to the CD-ROM condition among the total population (*P* = .04), women (*P* = .04), and the 35-49 year age group (*P* = .03), with effect sizes that can be categorized as small (among total population) to moderate (among women and 35-49 year age group). In addition, the print condition was rated as more personally relevant by the ≤ 34 year age group (*P* = .18) and the less educated respondents (*P* = .19), with moderate, though not statistically significant, effect sizes.

## Discussion

### Principal Results

The results of this study indicate that there are differences in the use and appreciation of a print-delivered versus CD-ROM-delivered, computer-tailored intervention. The differences were mainly in favor of the print-delivered intervention. The print feedback was read and saved more often than the CD-ROM feedback (some specific subgroups excepted), and the print feedback was perceived as more personally relevant in the total study group and in some of the subgroups, with small to moderate effect sizes.

Surprisingly, the print-delivered feedback was rated as more personally relevant. Personal relevance is considered to be a core characteristic and a potential working mechanism of computer-tailored interventions [16,17], and, in the present study, both interventions had the same level of personalization and individualization. Apparently, it is not only the feedback itself that is related to the perception of personal relevance, but also the delivery mode through which the information is distributed. Perhaps the immediate feedback on screen after completion of the questionnaire (in the CD-ROM condition) versus the time lag between returning the questionnaire to the researchers and receiving feedback (in the print condition) influences this perception. The receipt of a personalized mailed letter might also enhance relevance. Another explanation may be that participants had expected more personal relevance from a computer program in which they had to complete questions first.

### Comparison With Prior Work

Our study is unique in evaluating a broader set of indicators for use, appreciation, and perception of personal relevance between a print-delivered and an interactive-delivered, computer-tailored intervention with identical content.

The finding that the print-delivered feedback is read and saved more often than the CD-ROM-delivered feedback is in line with expectations and findings from previous studies [8,9,28,29].

Information sent through print media may be more easily available and accessible and easier to read and save [8,9]. Our results not only indicate that the subgroups suggested in the literature (women, less educated respondents, and older respondents) use the CD-ROM less than print, but also that this is the case for men and higher-educated and younger respondents. However, we do not know why participants in the CD-ROM group did not read the information. Having to use a computer and start a program may have been a barrier in terms of the time, effort, or planning that would be needed to use the program and generate the feedback. For another segment of the participants, lack of motivation or skills to use interactive media may have been a reason [13,21]. This could have been the case for women, older persons, and less-educated persons.

Vandelanotte et al found that people over 40 years compared to those younger than 40 years preferred an intervention delivered in print over an interactively delivered intervention [25]. However, it has also been found that even though people had indicated they preferred to receive an intervention over the Internet, they nevertheless did not access this intervention [29].

The findings of this study add to the evidence regarding differences in use of interactive and print-delivered interventions with identical content [28,29] and provide important new insights in appreciation and perceived relevance of the information. Findings from this and previous studies suggest that interactively delivered interventions as used to date may be less successful in attracting attention and may be less suited to facilitate active information processing compared to print-delivered computer-tailored information. Efforts are needed to increase use, appreciation, and active information processing.

### Limitations

The present study provides descriptive data. Further studies should explore if personal relevance and reading level mediate differential effects between print-delivered and interactive-delivered tailored feedback. Additionally, less-educated people and those older than 65 years were underrepresented or not included in this study. Although the intervention could be provided over the Internet, in this study it was delivered on a CD-ROM.

In this study we conducted a lot of tests without correction for multiple testing, which may increase the risk of false positives in the outcomes of the analyses. However, due to subgroup analyses, the number of participants was rather small in some analyses, which may have caused lack of power to detect significant differences, even when there was a moderate effect size. Reducing the *P* value to correct for multiple testing would increase the risk of false negatives. Therefore, we reported the uncorrected *P* values and the effect sizes of our different outcome measures. We evaluated the significance of differences using a significance level of *P* < .05. Effects can also be evaluated using a more conservative significance level of *P* < .01 to approach correction for multiple testing. In addition, the moderate effect sizes may provide an indication of differences that might become statistically significant when analyzed in larger groups.

Further, this study compared two delivery modes on aspects of use and appreciation that are relevant for both modes (ie, in both cases, for information processing, the information should be read, saved, and perceived as personally relevant). However, using this approach, we may have missed important aspects for use and probably appreciation of the information or the program that are more sensitive to specific characteristics of interactive media. Future process evaluation studies could use more extensive and specific instruments.

### Conclusions

Interactive computer-tailored feedback appears to be read and saved less than print-delivered feedback and perceived as less personally relevant, especially among certain subgroups. These differences in use and appreciation of the computer-tailored intervention delivered through print or interactive delivery modes can be taken into account when selecting a delivery mode for a specific subgroup in order to optimize exposure. Future studies should explore methods to improve exposure to and use of interactively delivered computer-tailored information.
